# Inflammation-Based C-Reactive Protein-to-Albumin Ratio for No-Reflow Prediction in STEMI

**DOI:** 10.3390/biomedicines14061383

**Published:** 2026-06-18

**Authors:** Xhevdet Krasniqi, Altinë Spanca, Gresa Gojani, Josip Vincelj, Blerim Berisha, Aurora Bakalli

**Affiliations:** 1 Department of Internal Medicine, Medical Faculty, University of Prishtina “Hasan Prishtina”, 10000 Prishtina, Kosovo; xhevdet.krasniqi@uni-pr.edu (X.K.); altine.spanca@student.uni-pr.edu (A.S.); gojanigresa@gmail.com (G.G.); 2 Department of Cardiology, University Clinical Center of Kosova, 10000 Prishtina, Kosovo; 3 Department of Cardiovascular Medicine, Dubrava University Hospital, HR-10000 Zagreb, Croatia; 4 Kreisklinik Roth, Kardiologie und Internistische Intensivmedizin, 91154 Roth, Bavaria, Germany

**Keywords:** CAR, no-reflow phenomenon, STEMI

## Abstract

**Background:** The C-reactive protein/albumin ratio (CAR) has increasingly attracted attention as a reliable predictive marker in patients with acute myocardial infarction (AMI). **Purpose:** This study aimed to evaluate the predictive value of the CAR for no-reflow development. **Methods:** A total of 201 patients with STEMI who underwent PCI were included in the study. Admission laboratory tests included CRP, albumin, CK, CK-MB, troponin T, and other biochemical parameters. The CAR was calculated as CRP divided by albumin ×100, while the CUAR was calculated as the base-10 logarithm of CRP × UA divided by albumin. Patients were then divided into two groups based on CAR levels. **Results:** A total of 201 STEMI patients were included: 106 (52.7%) in the low-CAR group (≤48.4) and 95 (47.3%) in the high-CAR group (>48.4). Significant differences between groups were observed for smoking, albumin, cholesterol, CRP, CUAR, and TIMI flow grade ≤ 2. Logistic regression analysis identified albumin, cholesterol, CRP, BUN, uric acid, CK-MB, CAR, and CUAR as significant predictors of TIMI flow grade. A receiver operating characteristic (ROC) curve of CRP, albumin, CAR, and CUAR was used to plot the true positive rate against the false positive rate across various cut-off points; the area under the curve (AUC) was 0.87 (95% CI, 0.81–0.94, *p* < 0.0001) for CRP, 0.73 (95% CI, 0.65–0.81, *p* < 0.0001) for albumin, 0.9 (95% CI, 0.84–0.95, *p* < 0.0001) for the CAR, and 0.94 (95%, 0.89–0.99, *p* < 0.0001) for the CUAR. The cut-off values were 2.11 for the CUAR, 48.4 for the CAR, 18 for CRP, and 38 for albumin. **Conclusions:** The ratio of C-reactive protein to albumin (CAR) may serve as a reliable and clinically accessible marker associated with the no-reflow phenomenon in STEMI patients undergoing PCI. A defined CAR cut-off has been proposed to help stratify patients at increased risk of no-reflow.

## 1. Introduction

ST elevation myocardial infarction (STEMI) continues to represent a leading cause of morbidity and mortality worldwide, despite significant advances in both pharmacological management and interventional cardiology. Timely reperfusion, most effectively achieved through primary percutaneous coronary intervention (PCI), remains the cornerstone of STEMI treatment, as it significantly limits infarct size and enhances patient survival [1]. However, the restoration of epicardial coronary artery patency does not always result in adequate myocardial perfusion.

The no-reflow phenomenon, characterized by inadequate myocardial microvascular perfusion despite successful recanalization of the infarct-related epicardial coronary artery, represents a well-recognized complication in patients with STEMI. This development reflects a dissociation between epicardial vessel patency and effective tissue-level reperfusion and is observed in a considerable proportion of STEMI cases, even following technically successful primary percutaneous coronary intervention [2].

The pathophysiology of the no-reflow phenomenon is complex and multifactorial, involving ischemia–reperfusion injury, endothelial dysfunction, distal embolization, and inflammation. These mechanisms act synergistically to induce microvascular damage, resulting in impaired myocardial perfusion despite successful restoration of epicardial coronary artery patency [3,4].

**Figure 1:**
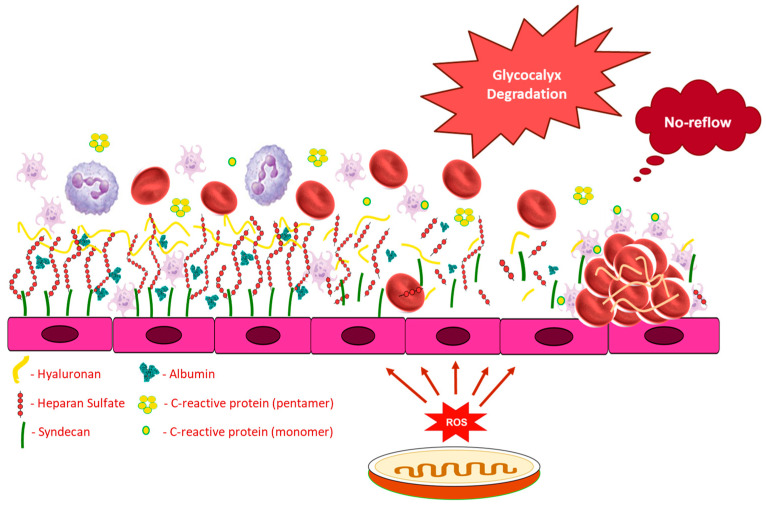
Endothelial glycocalyx degradation during ischemia–reperfusion injury.

Reactive oxygen species (ROS) play a central role in the degradation of the endothelial glycocalyx during myocardial ischemia–reperfusion injury. Excessive ROS generation—particularly during the early reperfusion phase—induces oxidative damage to the structural components of the glycocalyx, including proteoglycans (e.g., syndecans or glypicans) and glycosaminoglycans (e.g., heparan sulfate via the activation of sheddase enzymes such as matrix metalloproteinases and heparinase) (Figure 1) [5,6,7,8].

ROS induce oxidative modification of albumin, particularly through the oxidation of the free thiol group at cysteine-34, leading to the formation of oxidized albumin species with reduced antioxidant capacity and impaired ligand-binding properties. This functional alteration diminishes albumin’s protective role in maintaining oncotic pressure and scavenging free radicals, thereby exacerbating vascular and tissue injury [9,10,11].

Glycocalyx degradation promotes endothelial activation by exposing adhesion molecules, increasing leukocyte–endothelial interactions and sustaining inflammation with elevated cytokines and acute-phase reactants, including CRP. Beyond being a biomarker, CRP may further contribute to vascular injury through complement activation, oxidative stress, and ROS production. This creates a self-perpetuating inflammatory–oxidative cycle in which glycocalyx damage and CRP amplify endothelial dysfunction and microvascular injury, ultimately leading to distal microembolization and the no-reflow phenomenon [12,13,14], Figure 1.

Recently, the C-reactive protein/albumin ratio (CAR) has emerged as a novel inflammation-based biomarker that integrates CRP and albumin into a single index. This ratio provides a more comprehensive assessment of the balance between pro-inflammatory and anti-inflammatory processes. Several studies have demonstrated the prognostic value of the CAR in various cardiovascular conditions, including acute myocardial infarction, where it has been associated with increased mortality, complications, and adverse clinical outcomes [15].

Despite accumulating evidence supporting the prognostic significance of the CAR, its role in predicting the no-reflow phenomenon in STEMI patients remains incompletely elucidated. Previous studies have shown that the CAR is independently associated with the occurrence of no-reflow in patients with acute coronary syndromes, suggesting its potential utility as a simple and readily available risk stratification tool [16,17].

The novelty of the present study lies in proposing a mechanistic link between oxidative stress-induced endothelial glycocalyx degradation, inflammation, and the no-reflow phenomenon in STEMI, while positioning the C-reactive protein/albumin ratio (CAR) as a clinically accessible biomarker reflecting this inflammatory–oxidative microvascular injury axis.

## 2. Methods

### 2.1. Data Collection

The present investigation was based on retrospectively collected data. Relevant clinical data were obtained from hospital databases for consecutive STEMI patients treated with coronary angiography (CAG) and PCI during the period from January 2025 to December 2025. A total of 230 patients were screened, and 201 patients with a confirmed diagnosis of STEMI were included in the final analysis. A total of 29 patients were excluded from the analysis because CRP and albumin values were unavailable. Using a CAR cut-off value of 48.4, patients were categorized into two groups: group 1 (≤48.4) and group 2 (>48.4). STEMI eligibility required fulfillment of the following criteria: elevated cardiac troponin (cTn) based on ESC 0 h/1 h or 0 h/2 h algorithms; symptoms consistent with myocardial ischemia; new ST-segment elevation in at least two contiguous leads meeting established thresholds; and treatment with primary percutaneous coronary intervention (pPCI) within 12 h of symptom onset [1]. Patients with the following comorbidities during hospitalization were excluded from the study: myocardial injury secondary to oxygen supply–demand imbalance (such as aortic dissection and severe anemia); non-ischemic myocardial injury (including myocarditis and cardiotoxic chemotherapy); and multifactorial or indeterminate myocardial injury, including severe pulmonary embolism, sepsis, renal failure, stroke, and subarachnoid hemorrhage. A detailed clinical history was collected, including evaluation of established coronary risk factors (diabetes mellitus, dyslipidemia, arterial hypertension, and smoking), prior medication use, and the interval between symptom onset and hospital admission.

Blood samples were obtained upon admission, followed by comprehensive biochemical laboratory analyses. Specifically, cardiac biomarkers such as creatine kinase (CK), creatine kinase-MB (CK-MB), and cardiac troponin T (cTnT) were measured to assess myocardial injury. Furthermore, measurements of serum albumin, C-reactive protein (CRP), and lipid profile components (total cholesterol and triglycerides) were performed. The results of CRP and albumin measurements were available prior to PCI. Routine biochemical parameters were also evaluated as part of the standard laboratory workup. As in previous studies, the C-reactive protein-to-albumin ratio (CAR) was calculated by dividing serum CRP levels by serum albumin levels and multiplying the result by 100 to facilitate statistical analysis. Also, the CUAR was calculated as the base-10 logarithm of the product of C-reactive protein (CRP) and uric acid (UA), divided by albumin.

Revascularization was performed in all patients (Coronary angiography, Philips Allura Xper FD20 Cath/Angio System, Royal Philips, Best, The Netherlands), accompanied by a standardized regimen of antiplatelet and anticoagulant therapy, in accordance with current clinical guidelines. The door-to-balloon (DTB) time, representing the interval from hospital admission to balloon inflation during primary PCI, was less than 90 min. Onset-to-balloon (OTB) time, defined as the interval between the onset of myocardial infarction symptoms (STEMI) and balloon inflation during primary percutaneous coronary intervention (PCI), was less than 120 min. Antiplatelet therapy included the administration of acetylsalicylic acid (aspirin) with a loading dose (LD) of 150–300 mg administered orally, followed by a maintenance dose (MD) of 75–100 mg once daily (o.d.). In addition, a P2Y12 receptor inhibitor was administered: either clopidogrel with a loading dose of 300–600 mg orally followed by a maintenance dose of 75 mg once daily or prasugrel with a loading dose of 60 mg orally followed by a maintenance dose of 10 mg once daily. Anticoagulation during PCI was achieved with unfractionated heparin (70–100 U/kg i.v. bolus or adjusted according to ACT) [1,18]. Coronary perfusion was evaluated angiographically using the TIMI flow grading system (grades 0–3). The no-reflow phenomenon was defined as impaired epicardial flow (TIMI grade 0–2) after restoration of vessel patency. Final angiograms obtained immediately following primary PCI were independently assessed by two experienced interventional cardiologists blinded to the CRP/albumin ratio.

All patients underwent transthoracic echocardiographic evaluation with a Philips EPIQ 7C system (X5-1 probe; Philips Ultrasound LLC, Bothell, WA, USA) for the assessment of left ventricular ejection fraction (LVEF) and cardiac function.

This study adhered to the principles of the Declaration of Helsinki and received approval from the Ethics Committee of the Faculty of Medicine, University of Prishtina “Hasan Prishtina”.

### 2.2. Statistical Analysis

The present study was designed to determine whether the CRP/albumin ratio is associated with the occurrence of the no-reflow phenomenon in patients with STEMI. Continuous variables were assessed for normality and are presented as mean ± standard deviation (SD) for normally distributed data or median with interquartile range (IQR) for non-normally distributed data. Categorical variables are expressed as absolute numbers and corresponding percentages. Comparisons between groups were performed using Student’s t-test for normally distributed continuous variables and the Mann–Whitney U test for non-normally distributed variables. The chi-square test or Fisher’s exact test, as appropriate, was used for comparisons of categorical variables. Multivariable binary logistic regression analysis was then conducted to identify independent predictors of no-reflow. To avoid multicollinearity, CRP, albumin, and the CRP/albumin ratio were not entered simultaneously in the same model; the final model included the CRP/albumin ratio along with other clinically relevant covariates (e.g., age, sex, diabetes mellitus, and other relevant variables depending on the dataset). Results are reported as odds ratios (ORs) with corresponding 95% confidence intervals (CIs). The goodness-of-fit of the regression model was evaluated using the Hosmer–Lemeshow test. The discriminatory ability of studied variables was assessed using receiver operating characteristic (ROC) curve analysis. Sensitivity and specificity were plotted across a range of cut-off values, and the area under the curve (AUC) was calculated to determine predictive accuracy. The optimal cut-off value was defined as the point yielding the highest combined sensitivity and specificity.

All statistical tests were two-sided, and a *p*-value < 0.05 was considered statistically significant. Statistical analyses were performed using SPSS software (version 26.0).

## 3. Results

A comprehensive summary of the baseline characteristics of the study population is presented in Table 1. A total of 230 patients were screened, of whom 201 patients with STEMI were included in the study and subsequently stratified according to their C-reactive protein-to-albumin ratio (CAR). Of these, 106 patients (52.73%) were assigned to group 1 (CAR ≤ 48.4), while 95 patients (47.26%) comprised group 2 (CAR > 48.4). Comparative analysis between the two groups demonstrated statistically significant differences across several variables. Specifically, smoking status differed significantly between groups (*p* = 0.018). Marked differences were also observed in serum albumin levels and C-reactive protein (CRP) concentrations, both of which showed strong statistical significance (*p* < 0.0001 for both variables). In addition, total cholesterol levels were significantly different between the groups (*p* = 0.04). Furthermore, the no-reflow phenomenon, defined as a thrombolysis in myocardial infarction (TIMI) flow grade ≤ 2, differed significantly between the two groups (*p* < 0.0001). These findings are detailed in Table 1.

Binary logistic regression analysis was performed to evaluate the association between the dichotomous dependent variable (TIMI flow grade), and a set of independent clinical and biochemical variables, including hypertension, diabetes mellitus, smoking status, total cholesterol, triglycerides, blood urea nitrogen (BUN), creatinine, uric acid, creatine kinase-MB (CK-MB), total creatine kinase (CK), troponin T, C-reactive protein (CRP), serum albumin, C-reactive protein-to-albumin ratio (CAR), and the C-reactive protein/uric acid/albumin ratio (CUAR). The analysis identified several variables as statistically significant predictors at the 5% significance level. Specifically, serum albumin (*p* = 0.01), total cholesterol (*p* = 0.04), CRP (*p* < 0.0001), BUN (*p* = 0.02), uric acid (*p* = 0.039), CK-MB (*p* = 0.019), CAR (*p* < 0.0001), and CUAR (*p* < 0.0001) were independently associated with the outcome variable. These findings suggest that both inflammatory markers and metabolic parameters play significant roles in predicting impaired coronary perfusion, as reflected by TIMI flow grade (Table 2).

**Figure 2:**
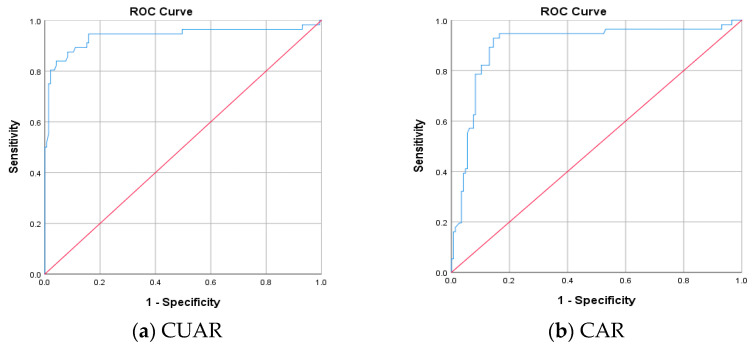

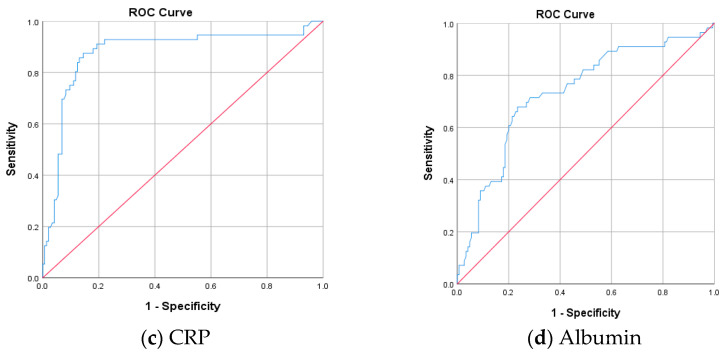
ROC curve analysis of CUAR, CAR, CRP, and albumin for the prediction of no-reflow phenomenon in STEMI patients.

A receiver operating characteristics (ROC) curve of CRP, albumin, and the CAR was used to plot the true positive rate against the false positive rate across varying cut-off points; the area under the curve (AUC) was 0.87 (95% CI, 0.81–0.94, *p* < 0.0001) for CRP, 0.73 (95% CI, 0.65–0.81, *p* < 0.0001) for albumin, 0.9 (95% CI, 0.84–0.95, *p* < 0.0001) for the CAR, and 0.94 (95%, 0.89–0.99, *p* < 0.0001) for the CUAR (Figure 2). The cut-off values were 2.11 for the CUAR, 48.4 for the CAR, 18 for CRP, and 38 for albumin.

Table 3 presents the area under the curve for biochemical analysis (creatinine, BUN, cholesterol, triglyceride, creatine kinase-MB, creatine kinase, troponin T, uric acid, CRP, glucose, albumin, CAR, and CUAR).

Table 4 presents the paired-sample differences in the areas under the ROC curves.

## 4. Discussion

The findings of the present study indicate that the C-reactive protein-to-albumin ratio (CAR) is an independent and clinically relevant predictor of the no-reflow phenomenon in STEMI patients undergoing primary PCI. The CAR was selected as the primary inflammatory marker because it integrates two biologically relevant components of the acute inflammatory response: C-reactive protein (CRP), a positive acute-phase reactant that increases during systemic inflammation, and albumin, a negative acute-phase reactant that decreases in inflammatory and catabolic states. Therefore, the CAR may more comprehensively reflect the balance between inflammation and nutritional or metabolic status compared with isolated inflammatory biomarkers [19,20]. It is noteworthy that the CAR demonstrated enhanced discriminative value relative to its individual components, CRP and albumin, suggesting that the integration of inflammatory and nutritional parameters yields superior prognostic performance. It may, therefore, improve risk stratification and enable early identification of patients at high risk of no-reflow in the setting of acute STEMI.

In addition to inflammatory pathways, glucometabolic stress may also play a significant role in the pathophysiology of the no-reflow phenomenon. In this context, the stress hyperglycemia ratio (SHR), a simple and readily available metabolic–inflammatory index, has recently been shown to be associated with periprocedural myocardial injury and infarction. Evidence suggests that the SHR may reflect acute metabolic stress responses that overlap with mechanisms implicated in no-reflow, including increased oxidative stress, endothelial dysfunction, and enhanced thrombotic burden [21].

The no-reflow phenomenon continues to represent a major challenge in interventional cardiology, manifesting even after successful reopening of the epicardial coronary artery. It is associated with larger infarct size, adverse left ventricular remodeling, heart failure, and increased mortality [22,23,24]. In our study, occurrence of no-reflow (TIMI flow grade ≤ 2) was significantly associated with inflammatory and metabolic parameters, supporting the concept that oxidative stress plays a pivotal role in myocardial ischemia–reperfusion injury, primary driven by the excessive production of reactive oxygen species upon restoration of coronary blood flow. This process leads to cellular damage, endothelial dysfunction, and activation of inflammatory cascades, ultimately leading to cardiomyocyte death and microvascular obstruction [25,26,27,28].

Our findings align with previous studies positioning CRP as a robust, independent biomarker of adverse clinical outcomes and no-reflow in acute myocardial infarction, highlighting the role of inflammation in microvascular dysfunction in reperfusion injury [13,29,30]. Beyond its role as a passive marker of systemic inflammation, CRP has also been implicated in the active modulation of endothelial dysfunction through complement activation, upregulation of adhesion molecules, and promotion of oxidative stress [31,32]. Serum albumin levels may decrease during acute myocardial infarction as a result of systemic inflammatory activation, since albumin behaves as a negative acute-phase reactant. Increased vascular permeability, cytokine-mediated suppression of hepatic synthesis, and oxidative stress may further contribute to reduced circulating albumin levels in this setting [33]. Alongside systemic inflammation, hypoalbuminemia reflects a state of impaired antioxidant defense capacity rather than merely a marker of nutritional status. Albumin is the most abundant circulating plasma protein and plays a critical biological role in maintaining redox balance through its ability to scavenge reactive oxygen species (ROS), bind transition metals, and limit lipid peroxidation. In addition, albumin contributes to endothelial homeostasis by preserving glycocalyx integrity, modulating vascular permeability, and exerting anti-inflammatory effects within the microvascular environment. Consequently, reduced serum albumin levels are associated with enhanced oxidative stress, endothelial dysfunction, and increased capillary leakage, all of which may contribute to microvascular injury and adverse outcomes in the ischemia–reperfusion setting, including the no-reflow phenomenon in acute myocardial infarction [9,34,35]. In this context, the CAR represents a composite index that captures pro-inflammatory activity and reduced antioxidant/anti-inflammatory capacity, thereby providing a more comprehensive assessment of net inflammatory burden compared with CRP or albumin alone. This explains why the CAR demonstrated the highest predictive performance in our study (AUC = 0.90), outperforming CRP and albumin separately (Figure 2).

The observed association between an elevated CAR and the no-reflow phenomenon likely reflects the integration of multiple interdependent pathophysiological processes inherent to ischemia–reperfusion injury. Excessive ROS generation induces endothelial glycocalyx degradation, increases vascular permeability, and promotes platelet aggregation and leukocyte adhesion. Simultaneously, elevated CRP may further amplify this injury through activation of the complement system and upregulation of inflammatory mediators, thereby exacerbating endothelial dysfunction. Concomitantly, hypoalbuminemia reflects both a heightened inflammatory state and a reduction in endogenous antioxidant capacity, limiting the neutralization of ROS and impairing vascular homeostasis. These mechanisms synergistically drive microvascular obstruction, distal embolization, and ultimately the no-reflow phenomenon despite successful epicardial reperfusion [36,37,38,39,40].

In addition, our binary logistic regression analysis identified several independent predictors of no-reflow, including CRP, albumin, CK-MB, uric acid, BUN, cholesterol, and the CAR. These findings highlight the multifactorial nature of microvascular obstruction, involving not only inflammation but also metabolic dysregulation, renal impairment, and the extent of myocardial necrosis [41,42,43]. This integrative pathophysiological framework is further supported by inclusion of the CAR, which reflects the balance between pro-inflammatory and anti-inflammatory/antioxidant states, thereby providing a more comprehensive representation of the inflammatory burden associated with no-reflow [44].

Importantly, ROC curve analysis demonstrated the superior predictive accuracy of the CAR compared with CRP and albumin individually. The CAR cut-off value of 48.4, identified within the present study population, may serve as a potentially useful parameter for early risk stratification in STEMI patients undergoing PCI. Given its simplicity and wide availability, the CAR could be readily implemented in routine clinical practice without additional cost or technical requirements; however, this threshold should be interpreted as exploratory and data-driven rather than a validated clinical cut-off. Accordingly, external validation in independent cohorts is required before any clinical application can be recommended.

Clinically, the early identification of patients at high risk of no-reflow is important as it may facilitate closer monitoring and improved risk stratification in the acute STEMI setting. Given its simplicity and wide availability, the CRP/albumin ratio (CAR) may represent a useful inflammatory marker for identifying patients at increased risk of no-reflow [16,45]. Although elevated CAR levels could potentially help identify patients who may benefit from adjunctive pharmacological or mechanical strategies aimed at improving microvascular perfusion, such as intracoronary vasodilators, thrombectomy, or intensified antithrombotic therapy, prospective interventional studies are required before the CAR can be implemented in routine clinical decision-making.

This study has several limitations. First, it is a single-center study with a relatively modest sample size, which may limit generalizability. Second, dynamic changes in the CAR during hospitalization were not assessed, which could provide additional prognostic information.

## 5. Conclusions

The present study indicates that the C-reactive protein-to-albumin ratio is associated with the no-reflow phenomenon in STEMI patients undergoing primary PCI. The CAR demonstrated better performance than its individual components and may serve as a simple, cost-effective, and clinically relevant biomarker for early risk stratification. Further large-scale, multicenter studies are warranted to validate these findings and to explore potential CAR-guided therapeutic strategies aimed at improving clinical outcomes.

## Figures and Tables

**Table 1:** Baseline characteristics of patients according to CAR level.

Characteristics	CAR ≤ 48.4 (n = 106)	CAR > 48.4 (n = 95)	*p*-Value
Age (year)	62.28 (±11.93)	64.11 (±11.79)	0.08
Gender (male) (n/%)	85 (80.28)	61 (64.21)	0.18
BMI (mean ± s.d.)	25.9 (±3.65)	28.13 (±6.12)	0.05
Hypertension (n/%)	66 (62.26)	62 (65.26)	0.78
DM (n/%)	37 (34.9)	39 (41.05)	0.56
Smoking (n/%)	56 (52.83)	40 (42.1)	0.018
Albumin (g/L)	40.14 (±5.9)	35.18 (±7.14)	<0.0001
Glucose (mmol/L)	8.67 (±4.67)	9.03 (±4.75)	0.15
Cholesterol (mmol/L)	4.74 (±1.22)	5.28 (±2.14)	0.04
Triglyceride (mmol/L)	1.99 (±1.06)	1.98 (±1.14)	0.97
Hemoglobin (mg/dL)	140.25 (±17.38)	147.29 (±19.58)	0.002
Creatinine (umol/L)	84.6 (30.0–145.56)	87.0 (43.30–196.4)	0.51
CRP (mg/L)	5.36 (0.3–70.20)	44.2 (1.7–232.0)	<0.0001
CUAR (mean ± s.d.)	1.67 (±0.58)	3.81 (±1.49)	<0.0001
BUN (mmol/L)	7.4.0 (±4.11)	9.44 (±7.7)	0.54
Creatine kinase-MB (U/L)	45.1 (49.0–300.0)	52.5 (62.0–685.0)	0.5
Creatine kinase (U/L)	298.5 (245.0–6138.0)	433.4 (311.0–6690.0)	0.54
Troponin T (pg/mL)	279.0 (31.03–4636.0)	293.45 (60.02–4850.0)	0.13
Killip class > 1 (n/%)	14 (13.2)	21 (22.1)	<0.0001
Ejection fraction (EF) (%)	50.21 (±9.1)	48.91 (±9.88)	0.56
Triple-vessel disease (n/%)	31 (26.96)	22 (27.63)	0.65
TIMI flow grade (≤2) (n/%)	9 (8.49)	47 (49.47)	<0.0001

BMI: Body mass index; DM: diabetes mellitus; CRP: C-reactive protein; CUAR: C-reactive protein/uric acid/albumin ratio; BUN: blood nitrogen urea; TIMI: thrombolysis in myocardial infarction.

**Table 2:** Binary logistic regression analysis for prediction of no-reflow phenomenon.

Parameter	OR	95% CI	*p*-Value
Hypertension (n/%)	1.13	0.51–2.49	0.75
DM (n/%)	0.58	0.27–1.25	0.16
Smoking (n/%)	1.35	0.66–2.73	0.4
Killip class > 1 (n/%)	0.16	0.06–0.4	<0.0001
Triple vessel disease (n/%)	0.4	0.11–1.4	0.15
Cholesterol (mmol/L)	3.47	1.04–11.59	0.04
Triglyceride (mmol/L)	0.39	0.11–1.3	0.12
Hemoglobin (mg/dL)	1.09	1.05–1.12	<0.0001
BUN (mmol/L)	1.14	1.04–1.26	0.05
Creatinine (umol/L)	0.99	0.97–1.0	0.22
Uric acid	1.01	0.96–1.0	0.039
Creatine kinase-MB (U/L)	1.12	0.9–0.99	0.019
Creatine kinase (U/L)	1.0	1.0–1.01	0.06
Troponin T (pg/mL)	1.0	0.99–1.0	0.32
CRP (mg/L)	1.06	1.04–1.07	<0.0001
Albumin (g/L)	1.08	1.01–1.15	0.01
CAR (median/range)	4.5	2.62–7.81	<0.0001
CUAR (mean ± s.d.)	4.84	3.02–7.74	<0.0001

DM: Diabetes mellitus; CRP: C-reactive protein; CAR: C-reactive protein/albumin ratio; CUAR: C-reactive protein/uric acid/albumin ratio.

**Table 3:** Area under the curve values for biochemical analysis.

Parameter	AUC (95% CI)	*p*-Value
Creatinine (umol/L)	0.57 (0.29–0.63)	0.41
BUN (mmol/L)	0.46 (0.21–0.62)	0.67
Cholesterol (mmol/L)	0.39 (0.24–0.55)	0.23
Triglyceride (mmol/L)	0.44 (0.26–0.62)	0.53
Creatine kinase-MB (U/L)	0.65 (0.48–0.81)	0.08
Creatine kinase (U/L)	0.54 (0.35–0.72)	0.62
Troponin T (pg/mL)	0.55 (0.45–0.65)	0.29
Uric acid (umol/L)	0.45 (0.26–0.63)	0.56
CRP (mg/L)	0.87 (0.81–0.94)	<0.0001
Glucose (mg/dL)	0.55 (0.46–0.63)	0.24
Albumin (g/L)	0.73 (0.65–0.81)	<0.0001
CAR (median/range)	0.9 (0.84–0.95)	<0.0001
CUAR (mean ± s.d.)	0.94 (0.89–0.99)	<0.0001

AUC: Area under the curve; BUN: blood urea nitrogen; CRP: C-reactive protein; CAR: C-reactive protein/albumin ratio; CUAR: C-reactive protein/uric acid/albumin ratio.

**Table 4:** Paired-sample area difference under the ROC curves.

Pair(s)	Δ AUC	*p*-Value
Albumin–CRP	−0.61 (−0.72 to −0.50)	<0.0001
Albumin–CAR	−0.63 (−0.74 to −0.52)	<0.0001
Albumin–CUAR	−0.67 (−0.77 to −0.57)	<0.0001
CRP–CAR	−0.02 (−0.051 to −0.009)	0.17
CRP–CUAR	−0.05 (−0.09 to −0.02)	0.001
CAR–CUAR	−0.038 (−0.063 to −0.014)	0.002

AUC: Area under the curve; CRP: C-reactive protein; CAR: C-reactive protein/albumin ratio; CUAR: C-reactive protein/uric acid/albumin ratio.

## Data Availability

The data presented in this study are available from the corresponding author upon reasonable request due to privacy and ethical considerations.
